# An Aptamer-Based Lateral Flow Biosensor for Low-Cost, Rapid and Instrument-Free Detection of Ochratoxin A in Food Samples

**DOI:** 10.3390/molecules28248135

**Published:** 2023-12-17

**Authors:** Electra Mermiga, Varvara Pagkali, Christos Kokkinos, Anastasios Economou

**Affiliations:** Department of Chemistry, National and Kapodistrian University of Athens, 157 71 Athens, Greece; electram24@gmail.com (E.M.); bpagali@yahoo.gr (V.P.); christok@chem.uoa.gr (C.K.)

**Keywords:** ochratoxin A, aptamer, aptasensor, biosensor, gold nanoparticles, lateral flow, colorimetry

## Abstract

In this work, a simple and cost-efficient aptasensor strip is developed for the rapid detection of OTA in food samples. The biosensor is based on the lateral flow assay concept using an OTA-specific aptamer for biorecognition of the target analyte. The strip consists of a sample pad, a conjugate pad, a nitrocellulose membrane (NC) and an absorbent pad. The conjugate pad is loaded with the OTA-specific aptamer conjugated with gold nanoparticles (AuNPs). The test line of the NC membrane is loaded with a specific OTA-aptamer probe and the control line is loaded with a control probe. The assay is based on a competitive format, where the OTA present in the sample combines with the OTA aptamer-AuNP conjugate and prevents the interaction between the specific probe immobilized on the test line and the OTA aptamer-AuNP conjugates; therefore, the color intensity of the test line decreases as the concentration of OTA in the sample increases. Qualitative detection of OTA is performed visually, while quantification is performed by reflectance colorimetry using a commercial scanner and image analysis. All the parameters of the assay are investigated in detail and the analytical features are established. The visual limit of detection (LOD) of the strip is 0.05 ng mL^−1^, while the LOD for semi-quantitative detection using reflectance colorimetry is 0.02 ng mL^−1^. The lateral flow strip aptasensor is applied to the detection of OTA in wine, beer, apple juice and milk samples with recoveries in the range from 91 to 114%. The assay exhibits a satisfactory selectivity for OTA with respect to other mycotoxins and lasts 20 min. Therefore, the lateral flow strip aptasensor could be useful for the rapid, low-cost and fit-for-purpose on-site detection of OTA in food samples.

## 1. Introduction

Ochratoxin A (OTA) is a mycotoxin produced as a secondary metabolite by several fungal species such as *Aspergillus* and *Penicillium* [1,2]. Various studies have shown that OTA can cause several adverse health effects to animals and humans through the consumption of contaminated agricultural goods, such as cereal grains, vegetables, coffee beans, wine and beer [2,3]. OTA has been shown to be nephrotoxic, teratogenic, immunotoxic and carcinogenic and therefore the International Agency for Research on Cancer (IARC) has classified OTA as a group 2B possible human carcinogen [4]. Due to the toxicity of OTA, the European Union has set maximum limits (MLs) for OTA in foods in the range of 0.5–10 μg kg^−1^ [4].

Considering the severe toxic effects and the low permitted MLs of OTA in food, it is of great importance to develop rapid and sensitive sensing platforms for OTA monitoring to address issues of food safety and avoid or minimize the risk of OTA intake by consumers. The detection of OTA in food is mostly based on conventional chromatographic techniques such as thin-layer chromatography (TLC), high-performance liquid chromatography (HPLC) or gas chromatography (GC), which, although powerful, require expensive and bulky equipment, trained personnel and a complex sample preparation and are not suitable for on-site rapid assays [2,4,5]. On the contrary, immunochromatographic (lateral flow) assays, utilizing antibodies as biorecognition elements, are convenient, portable, cost-effective and simple-to-use analytical and diagnostic platforms, providing satisfactory sensitivity with the potential for on-site rapid screening by non-trained personnel [6,7]; however, these devices require the use of expensive antibodies which are available for only a limited spectrum of target analytes and possess low chemical stability [8,9,10,11,12].

Aptamers have attracted the interest of the scientific community since their discovery in 1990 [13,14]. Aptamers are short single-stranded oligonucleotides (DNA or RNA) which can interact with a wide range of target analytes, such as antibiotics, proteins or other organic compounds [15,16]. Aptamers are selected from large synthetic DNA or RNA libraries, through a process called SELEX (Systematic Evolution of Ligands by EXponential enrichment). Through the SELEX process, it is possible to identify specific DNA sequences which bind to the target analyte(s) with high specificity by exploiting conformational changes in the three-dimensional structure of the oligonucleotides [13,14,15,16,17]. When serving as biorecognition elements, aptamers possess several advantages over antibodies, including their low cost, facile artificial synthesis, wider potential target range, good stability, resistance to heating and long shelf-life; these advantages make them ideal for rapid on-site detection and the development of inexpensive and portable analytical devices [13,14,15,16,17,18,19]. Over the last few years, aptamer-based sensors with optical or electrochemical detection have been used to detect a wide range of analytes [19,20], including OTA [4,12].

The aim of this work is the development of a simple, portable, instrument-free and cost-efficient lateral flow strip aptasensor for OTA determination in foodstuffs with improved analytical features over existing sensors. The biosensor strip is based on a competitive lateral flow assay principle using a conjugate of an OTA-specific aptamer with gold nanoparticles (AuNPs) as the biorecognition element. The test line of the strip contains a specific OTA-aptamer probe. In the presence of OTA, the OTA aptamer-AuNP conjugate is bound by the target analyte and is not allowed to bind with the specific probe of the test line in the strip. Qualitative detection of OTA is performed visually while quantification is performed by reflectance colorimetry using a scanner and image analysis. All the parameters of the assay are investigated in detail to provide optimum assay performance.

## 2. Results and Discussion

### 2.1. Assembly and Working Principle of the Lateral Flow Aptasensor 

**Figure 1:**
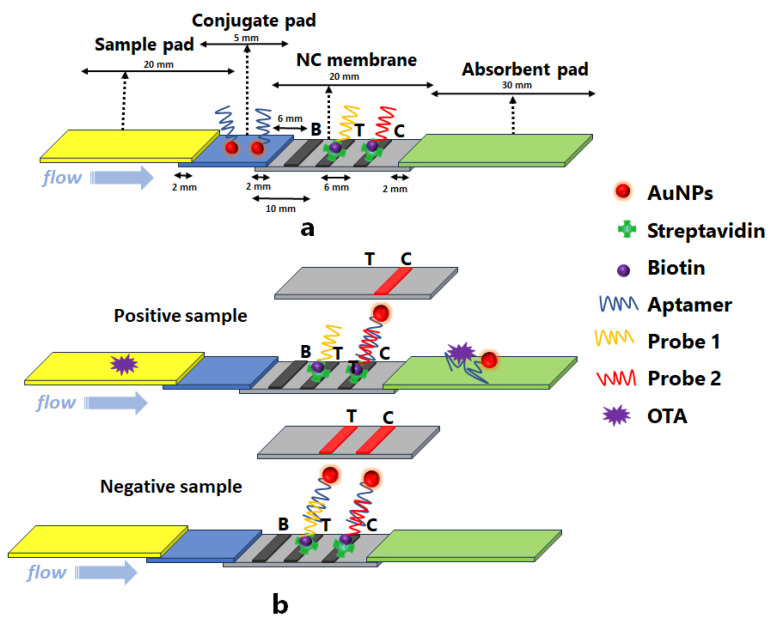
(**a**) Structure of the lateral flow strip and (**b**) principle of lateral flow immunoassay (dimensions are not drawn to scale). B is the PVA barrier, T is the test line and C is the control line.

A schematic of the lateral flow strip with dimensions is illustrated in Figure 1a. It consists of four successively placed segments: a sample pad; a conjugate pad; a NC membrane; and an absorbent pad, all with a width of 3 mm and lengths of 20 mm, 5 mm, 20 mm and 30 mm, respectively. The components mutually overlap by 2 mm to ensure continuous liquid flow.

The principle of the aptamer-based lateral flow strip for the detection of OTA is illustrated in Figure 1b. It is based on the competition between probe 1 immobilized on the test line and the OTA target present in the sample to combine with the OTA-specific aptamer that is conjugated with AuNPs, loaded onto the conjugate pad. If OTA is present in the sample, it will bind to the aptamer-AuNP conjugate, preventing the interaction between the aptamer-AuNP conjugate and probe 1 at the test line. If OTA is not present in the sample, the aptamer-AuNP conjugate will be available to interact with, and bind to, probe 1 at the test line. Therefore, the intensity of the test line decreases as the concentration of OTA in the sample increases. To ensure the validity of the test, the aptamer-AuNP conjugate interacts with control probe 2 at the control line to provide a visual signal regardless of whether or not OTA is present in the sample.

### 2.2. Optimization of Experimental Parameters

In order to achieve the best analytical performance of the developed assay, it was necessary to study various experimental parameters such as the signal processing method used for quantification; the aptamer sequence; the preparation of the OTA aptamer-AuNP conjugate; the method of AuNP preparation; the concentrations of streptavidin and of probe 1 and probe 2 immobilized at the test and control line, respectively; the selection of the appropriate NC membrane, the conjugate pad and the sample pad; the volume of the OTA aptamer-AuNP conjugate loaded onto the conjugate pad; the volume of sample applied at the sample pad; and the composition of the running buffer. The selection of the experimental conditions was based on the blank solution (which gives the maximum signal intensity) as well as on the assay sensitivity as determined by the % color intensity inhibition values (with respect to the intensity of the blank solution) or the color intensity values corresponding to signals of standard solutions.

**Figure 2:**
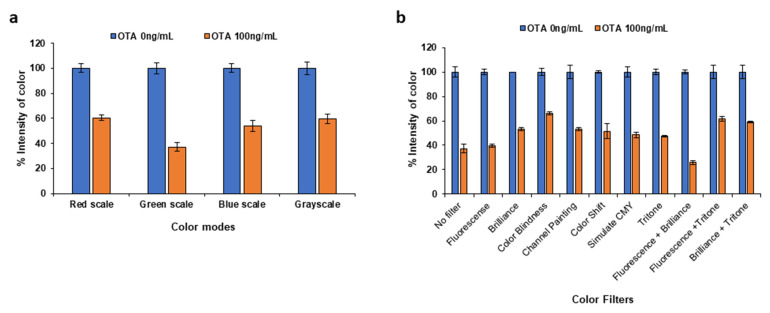
Effect of (**a**) the color mode (grayscale and RGB) detection and (**b**) the filters applied on the analytical signal.

Initially, the influence of the color mode detection was evaluated. The color intensities at the test and control lines were converted to grayscale and RGB color modes by using Inkscape. As shown in Figure 2a, when the color intensity was measured by reading the green component, the sensitivity of detection was highest. In addition, the effect of different color filters was also examined. As demonstrated in Figure 2b, the combination of fluorescence and brilliance filters led to a greater difference between the blank and the OTA standard. Therefore, the processing of all the colorimetric scanned images was performed using the green color with the application of both fluorescence and brilliance filters.

**Figure 3:**
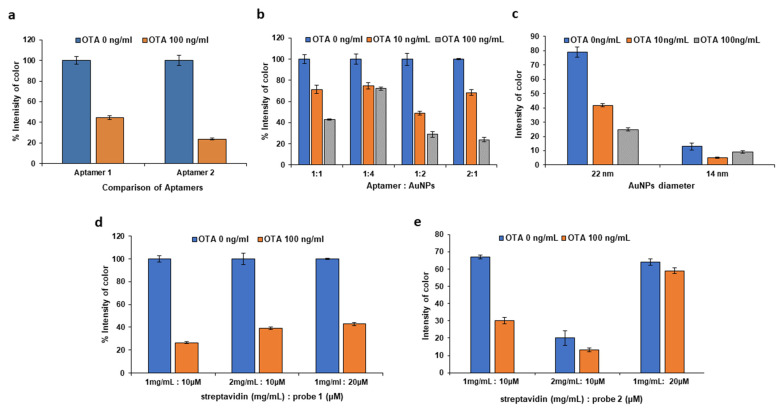
Effect of (**a**) the two OTA-specific aptamers (aptamer 1 and aptamer 2), (**b**) the volume ratio of the aptamer to AuNPs for the formation of OTA aptamer-AuNP conjugate, (**c**) the AuNP diameter, (**d**) the concentration of streptavidin and probe 1 for the test line and (**e**) the concentration of streptavidin and probe 2 for the control line.

Then, a comparison of two OTA-thiol-modified specific aptamers (aptamer 1: 5′/-thiolMC6-D/**GAT CGG GTG TGG GTG GCG TAA AGG GAG CAT CGG ACA** AAA AAA AAA AAA AAA AAA-3′ and aptamer 2: 5′/-thiolMC6-D/AAA AAA AAA AAA AAA AAA **GAT CGG GTG TGG GTG GCG TAA AGG GAG CAT CGG ACA**-3′) was carried out. The active part of the two aptamers (denoted by the sequence in bold) is the same as those of the two aptamers only differing in the positioning of the poly-A chain which is located close to 3′ and 5′ end of the aptamers, respectively. As shown in Figure 3a, the assay sensitivity is greatly improved with aptamer 2 and, therefore, this is selected for further investigation.

The volume ratio of the aptamer to AuNPs for the formation of the aptamer-AuNP conjugate was further studied in the range from 1:4 to 2:1; the 1:2 volume ratio of aptamer to AuNPs exhibited the highest color intensity changes and better differentiation among the OTA concentrations (Figure 3b).

AuNPs were prepared according to two different methods, the modified Frens method and the Turkevich method, with particle diameters of 22 and 14 nm, respectively. The AuNPs with a diameter of 22 nm, prepared with the modified Frens method, yielded higher signal intensities (Figure 3c).

Another important parameter is the concentration of streptavidin and of probes 1 and 2 during the preparation of streptavidin–biotin–probe conjugates. When the concentration of streptavidin was 1 mg mL^−1^ and the concentration of probe 1 at the test line was 10 μmol L^−1^, the addition of an OTA standard caused the most effective decrease in the signal of the test line, thus improving the assay sensitivity (Figure 3d). As for probe 2 at the control line, the addition of OTA should not affect the intensity of the control line; as illustrated in Figure 3e, the effect of OTA was minimal and the intensity of the control line was highest at 1 mg mL^−1^ of streptavidin and 20 μmol L^−1^ of probe 2.

Among different types of membranes, NC membranes are the most suitable for lateral flow assays. An important parameter for the selection of the NC membrane is the solution capillary flow rate. Two commonly used NC membranes were tested, Immunopore FP and Immunopore RP membrane, with flow rates of 140–200 s/4 cm and 90–150 s/4 cm, respectively. As shown in Figure S2a (Supplementary Information), when the Immunopore RP membrane was used, the addition of the OTA caused a more significant decrease in the signal of the test line.

As for the conjugate pad, two types of glass fiber pads (Whatman Standard 17, glass fiber cellulose pads) were investigated and the results (Figure S2b, Supplementary Information) indicated that Whatman Standard 17 resulted in a lower color intensity in the test line when OTA was introduced in the strip.

Four types of sample pad (Whatman Standard 17, Whatman Chromatography Paper No. 1, Whatman Chromatography Paper 3MM, Cellulose Fiber Sample Pads) were tested. The pad that proved most suitable for the strip was Whatman chromatography paper 3MM (Figure S2c, Supplementary Information), as it enhanced the sensitivity of the assay by ensuring a homogenous transportation of the sample and prevented the strip from overflowing.

**Figure 4:**
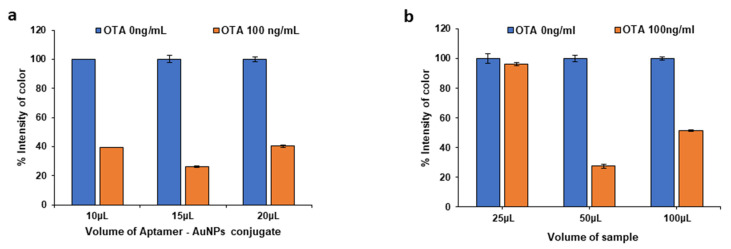
Selection of (**a**) the volume of the OTA aptamer/AuNP conjugate applied on the conjugate pad and (**b**) the volume of sample added on the sample pad.

Another important parameter is the volume of the AuNP-aptamer conjugate and the volume of sample loaded on the conjugate and sample pads. Varying volumes (5–20 µL) of the conjugate were loaded on the conjugate pad and the results are shown in Figure 4a. Using low volumes of the conjugate (<10 µL) resulted in lower test line intensities and the signal obtained for the OTA standard was not distinguishable from the blank signal. An amount of 15 µL of the OTA aptamer-AuNP conjugate solution provided the highest sensitivity and was selected for subsequent studies.

Regarding the volume of the sample on the sample pad, when 25 μL of sample was applied, the flow towards the NC membrane was not uniform and continuous; thus, the signal of the OTA standard did not differ from the blank signal. Conversely, the addition of 100 μL of sample caused the sample pad to overflow, leading to a loss of analyte and consequently to the decrease in assay sensitivity. As shown in Figure 4b, the most suitable sample volume was 50 μL as this produced the maximum signal difference between the blank signal and the signal due to the OTA standard.

**Figure 5:**
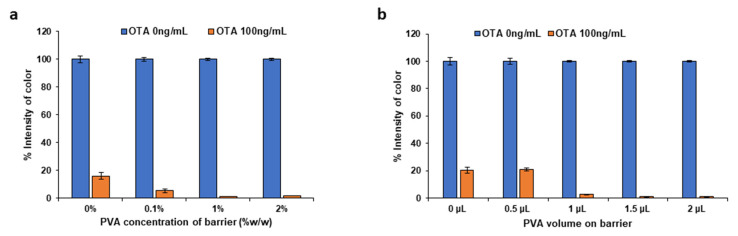
Effect of (**a**) the concentration and (**b**) the volume of PVA used to form PVA barrier.

In order to control the sample flow rate and increase the interaction time between the target analyte and the aptamer-AuNP conjugates, a PVA flow barrier was introduced into the NC membrane upstream of the test line. The PVA barrier contributes to enhanced sensitivity by delaying the sample flow, until it gradually dissolves, as the sample passes through the NC membrane pores [21,22]. In this study, by using a PVA barrier, the flow of analyte was effectively slowed down, leading to an increased biorecognition interaction time between the OTA in the sample and the aptamer-AuNP conjugates. Different concentrations of PVA (0–2% *w*/*w* PVA) (Figure 5a) and volumes of PVA (0–2.0 μL) (Figure 5b) were tested. As shown in Figure 5a, as the PVA concentration was increased, the sensitivity improved up to 1% *w*/*w* PVA. The signal was also enhanced by increasing the volume of the PVA solution placed on the NC membrane (Figure 5b); a volume of 1.5 μL of 1% *w*/*w* PVA solution was finally selected. The results indicate that using a barrier with 1% *w*/*w* PVA compared to a strip without a PVA barrier improved the visual LOD from 1 ng mL^−1^ to 0.05 ng mL^−1^, at the expense of a higher assay time.

To improve the sensitivity of detection, the running buffer composition is an important factor; thus, several types of running buffers were tested. Initially, four basic running buffers were tested containing 2% *w*/*w* sucrose, 1% *v*/*v* Tween 20, 0.02% *w*/*w* MgSO_4_, 0.05% *w*/*w* (NH_4_)_2_SO_4_ and 1% *w*/*w* BSA as common ingredients, with the addition of a. 50 mmol L^−1^ PBS (pH 7.4), b. 200 mmol L^−1^ tris-HCI buffer (pH 8.8), c. 50 mmol L^−1^ sodium citrate buffer (pH 6.5), and d. 50 mmol L^−1^ sodium borate buffer (pH 8.5). The use of the tris-HCI buffer and the sodium citrate buffers led to aggregation of the nanoparticles, as the color of the OTA aptamer-AuNP conjugates turned purple. Comparing the PBS and the sodium borate buffer, the results indicated that a higher sensitivity was observed with the PBS (Figure S3a, Supplementary Information).

Another important factor towards sensitivity enhancement is the addition of macromolecular aggregating agents to the running buffer. The influence of such reagents on assay sensitivity has been previously demonstrated on lateral flow immunoassays [23]. In this work, we studied how macromolecular crowding agents affect the sensitivity of the lateral flow aptasensor using PEG 20000, Ficoll F-400 and Ficoll F-70. The addition of PEG at various concentrations in the running buffer resulted in the appearance of a strong non-specific signal (Figure S3b, Supplementary Information). On the contrary, the addition of Ficoll, especially Ficoll 70, improved the assay sensitivity. Comparing F-400 and F-70, F-70 caused a more drastic decrease in signal intensity among the different OTA concentrations (Figure S3c, Supplementary Information). As shown in Figure S3d (Supplementary Information), the optimum concentration of F-70 was found to be 0.1% *w*/*w*, while concentrations higher than 0.1% *w*/*w* led to an increase in both the specific and the non-specific signal. The addition of F-70 contributed to the sensitivity enhancement, but most importantly increased the signal intensity of the control line by 45%. Another essential component of the running buffer is sucrose, in accordance with earlier reports [24]. The addition of 2% *w*/*w* sucrose to the running buffer resulted in the maximum signal intensity of the blank without increasing the non-specific signal of the OTA standard (Figure S4a, Supplementary Information). The effect of different surfactants (Tween 20, Triton X-114, SDS), proteins (BSA) and other compounds (PVP) in the running buffer was investigated. As shown in Figure S4 (Supplementary Information), the best composition for the running buffer was found to be 0.1% *w*/*w* Ficoll F-70, 2% *w*/*w* sucrose, 1% *v*/*v* Tween 20, 0.02% *w*/*w* MgSO_4_, 0.05% *w*/*w* (NH_4_)_2_SO_4_ and 1% *w*/*w* BSA in 50 mmol L^−1^ PBS (pH 7.4).

### 2.3. Analytical Performance

**Figure 6:**
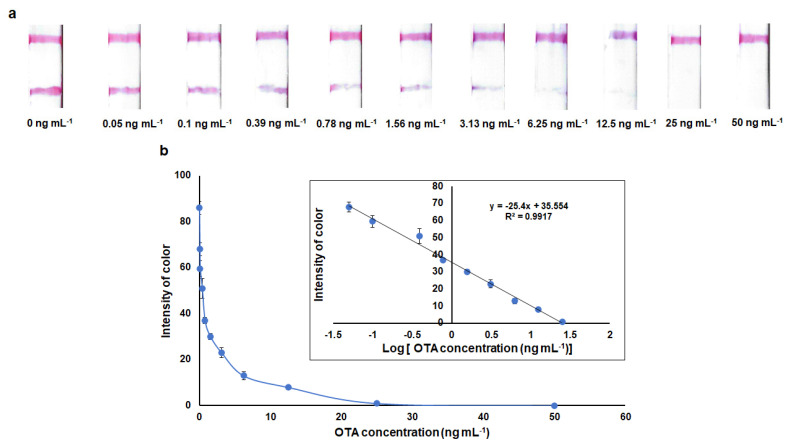
(**a**) Scanned images of the strips at different OTA concentrations in the range of 0–50 ng mL^−1^, (**b**) calibration curve for OTA (the inset shows the linear relationship between the signal of the test line and the logarithm of the OTA concentration).

Based on the selected detection conditions, the sensitivity, the specificity and the stability of the lateral flow assay for OTA were assessed. Standard solutions of OTA at various concentrations (0 to 50 ng mL^−1^) were prepared by diluting the OTA stock solution in running buffer and each standard was measured in triplicate. Typical scanned images of the strips using various concentrations of OTA are shown in Figure 6a. It can be seen that the blank solution produced well-defined test and control lines while the color intensity at the test lines decreased as the OTA concentration increased from 0 ng mL^−1^ to 50 ng mL^−1^. For OTA concentration ≥ 0.05 ng mL^−1^, distinguishable differences in the test line intensity were visually observed compared to the intensity of the blank, while the test line disappeared when the OTA concentration was >25 ng mL^−1^. According to the definition of the visual limit of detection (LOD)—the minimum concentration of OTA leading to the color of the test line to be visually identified as weaker than the color of the control line [25]—the visual LOD of the aptamer-based strip was considered to be 0.05 ng mL^−1^_._ The quantitative LOD was calculated from the calibration curve (Figure 6b) as 0.02 ng mL^−1^. The within-day assay reproducibility (expressed as the average % RSD of color intensities across the entire calibration range calculated from triplicate assays at each calibration level in a single day) was 5.5%. The between-day assay reproducibility (expressed as the average % RSD of color intensities across the entire calibration range calculated from triplicate assays at each calibration level over three different days) was 8.2%.

**Figure 7:**
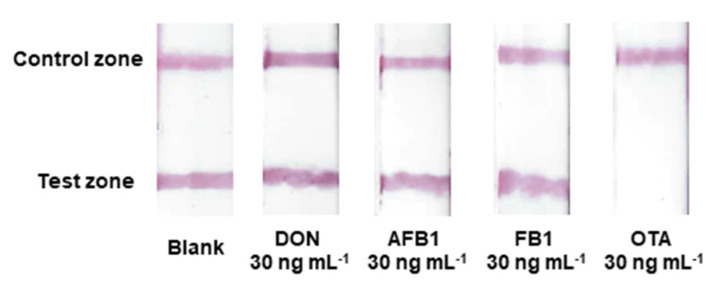
Selectivity study of the lateral flow assay.

The specificity of the aptamer-based test strip is of great importance and was confirmed by studying potential interferences from three common mycotoxins with a similar structure to OTA: deoxynivalenol (DON), aflatoxin B1 (AFB1) and fumonisin B1 (FB1). These mycotoxins displayed a negligible influence on the test lines at equal concentrations (meaning that the average signal intensities of the control line in the presence of interferents were within ±5% of the blank signal), indicating a negligible cross-reactivity to other common mycotoxins and a good specificity of the strip for the detection of OTA (Figure 7).

**Figure 8:**
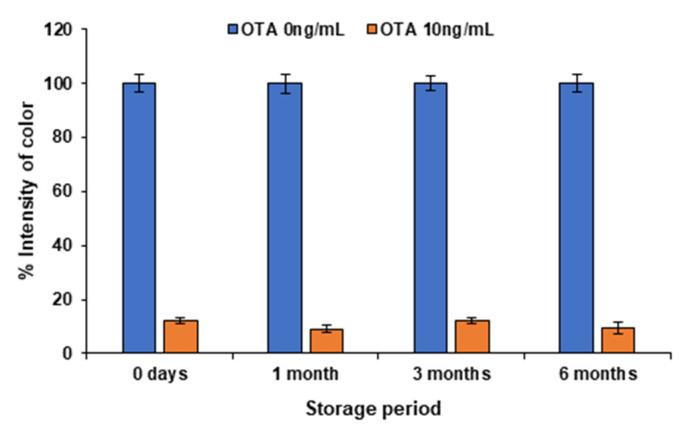
Stability of test of the lateral flow aptasensor.

In order to verify the stability of the aptasensor, strips from the same batch were prepared and stored at 4 °C for one month, three months and six months, respectively. As illustrated in Figure 8, there was no statistical difference between the signal of the test line at the strips stored for one, three and six months. Hence, the stability of the lateral flow strip remained satisfactory after a storage period of 6 months.

### 2.4. Application of the Lateral Flow Aptasensor to Food Samples

As a way to evaluate and verify the applicability and the accuracy of the developed aptamer-based LFA, samples spiked with OTA at specific concentrations (0.1, 1, 10 ng mL^−1^) were prepared by spiking appropriate volumes of a 2 μg mL^−1^ standard OTA solution in OTA-free beer, wine, apple juice and milk samples. The samples were analyzed in triplicate and the results are shown in Table 1. The recoveries ranged from 95 to 105%, 91 to 109%, 95 to 114% and 91 to 113% for beer, wine, apple juice and milk samples, respectively, and the % relative standard deviations (% RSDs) were in the range from 3.4 to 11.7 for beer, 3.3 to 11.8 for wine samples, 2.2 to 4.4 for apple juice and 3.3 to 13.3 for milk samples, demonstrating that the aptasensor had satisfactory accuracy and precision for OTA detection in various samples. It should be pointed out that, to the best of our knowledge, this is the first time that OTA has been effectively detected in spiked apple juice and milk samples using a lateral flow biosensor.

The analytical performance of the developed lateral flow strip was further confirmed by comparing the analytical and operational features with those of other validated aptamer-based lateral flow assays for OTA detection (Table 2). The aptasensor developed in this work exhibits better analytical features than existing colorimetric, visual and fluorometric lateral flow aptasensors at comparable assay times using a simple assay protocol. Only one work has reported better analytical characteristics than the current work, but this method makes use of a very complicated assay protocol, requires additional reagents, is less cost-effective and is more time consuming [26]; therefore, it is less suitable for on-site rapid screening purposes.

## 3. Materials, Equipment and Methods

### 3.1. Reagents and Materials

Ochratoxin A (OTA), aflatoxin B1 (AFB1), fumonisin B1 (FB1), deoxynivalenol (DON), streptavidin, sucrose, polysorbate 20 (Tween-20), polyethylene glycol (PEG 20000), polyvinylpyrrolidone (PVP), poly vinyl alcohol (PVA), potassium carbonate (K_2_CO_3_), sodium hydroxide (NaOH), sodium tetraborate decahydrate (Na_2_B_4_O_7_·10H_2_O), tetrachloroauric (III) acid trihydrate 99% (HAuCl_4_·3H_2_O, ≥99.9%) and boric acid (H_3_BO_3_) were purchased from Sigma-Aldrich (St. Louis, MO, USA). Nitric acid (HNO_3_) 65%, trisodium citrate dihydrate (C_6_H_5_Na_3_O_7_·2H_2_O), sodium chloride (NaCl), disodium hydrogen phosphate (Na_2_HPO_4_) potassium chloride (KCl) and monobasic potassium phosphate (KH_2_PO_4_) were obtained from Chem-Lab NV (Zedelgem, Belgium). Hydrochloric acid (HCl) 30%, magnesium sulfate heptahydrate (MgSO_4_·7H_2_O) and ammonium sulfate (NH_4_)_2_SO_4_ were obtained from Merck (Darmstadt, Germany). Tris (hydroxymethyl) aminomethane (C_4_H_11_NO_3_) was obtained from Duchefa Biochemie (Haarlem, The Netherlands), bovine serum albumin (BSA) was obtained from ThermoFisher Scientific (Waltham, MA, USA) and sodium dodecyl sulfate (SDS) was obtained from Fluka Biochemika (Buchs, Switzerland). Ficoll F-400 (Mr 400,000) was purchased from Sigma-Aldrich, while Ficoll F-70 (Mr 70,000) was obtained from Santa Cruz Biotechnology (Dallas, TX, USA). Water for molecular biology was supplied by PanReac AppliChem (Darmstadt, Germany). All other inorganic chemicals and organic solvents were of analytical grade. All the solutions were prepared with ultrapure water (resistivity 18.2 MΩ·cm).

Nitrocellulose (NC) membranes (Immunopore FP 5 μm, 25 mm × 50 m, 140–200 s/4 cm; Immunopore RP 8 μm, 25 mm × 50 m, 90–150 s/4 cm), Whatman STANDARD 17 bound glass fiber (34.5 s/4 cm), Whatman 3 MM Chromatography Paper and Whatman Chromatography Paper No. 1 were obtained from Whatman (Kent, UK). Glass fiber diagnostic pads and cellulose fiber sample pads were purchased from Merck Millipore (Burlington, MA, USA). HPLC-certified disposable syringe filters (pore size 0.20 μm) were obtained from MACHEREY-NAGEL (Duren, Germany)

Thiol-modified aptamers specific to OTA and biotin-modified DNA probes were obtained from Integrated DNA Technologies (Coralville, IA, USA). The detailed sequence of the aptamers and DNA probes were as follows:

Aptamer 1: 5′/-thiolMC6-D/GAT CGG GTG TGG GTG GCG TAA AGG GAG CAT CGG ACA AAA AAA AAA AAA AAA AAA-3′.

Aptamer 2: 5′/-thiolMC6-D/AAA AAA AAA AAA AAA AAA GAT CGG GTG TGG GTG GCG TAA AGG GAG CAT CGG ACA-3′.

Probe 1: 5′-/5biosg/TGT CCG ATG CTC CCT TTA CGC CAC CCA CAC CCG ATC-3′ (Test Line).

Probe 2: 5′-/5biosg/TTT TTT TTT TTT TTT TTT-3′ (Control Line).

In addition, 100 μmol L^−1^ stock solutions of the two aptamers and the complementary strands (probe 1 and probe 2) were prepared by dissolving the as-received compounds with molecular-biology-grade water and storing at −20 °C. An amount of 10 µmol L^−1^ of the aptamer solutions was prepared by adding 20 µL of the 100 μmol L^−1^ stock solutions to 180 µL of molecular-biology-grade water. A 10 μmol L^−1^ solution of probe 1 was prepared by diluting 3.5 µL of the 100 μmol L^−1^ stock solution to a final volume of 35 µL with molecular-biology-grade water. A 20 μmol L^−1^ solution of probe 2 was prepared by adding 7 µL of the 100 μmol L^−1^ stock solution to 28 µL with molecular-biology-grade water.

A 1 mg mL^−1^ stock OTA solution was prepared by dissolving 2 mg of OTA in 2 mL of methanol and stored in aliquots at −20 °C. More dilute working OTA solutions were prepared by dilution of the stock solution in the running buffer.

### 3.2. Equipment

A 10 μL syringe (Agilent Technologies, Santa Clara, CA, USA) was used to immobilize the reagents of the test and the control lines on the NC membrane. The Vortex mixer (VM + 10), table-top centrifuge (Eppendorf CF-10), hotplate magnetic stirrer (MSH-20D) and ultrasonic bath were supplied by Witeg (Wertheim, Germany). The analytical balance (220 g capacity, 0.1 mg readability) was obtained from Radwag (Radom, Poland), while a UV/Vis spectrophotometer (UV-1800) was obtained from Shimadzu (Kyoto, Japan)).

Optical detection on the lateral flow strips was based on reflectance colorimetry. The strips were scanned with an HP Deskjet 2720 scanner (Palo Alto, CA, USA) using 1200 dpi resolution. The further processing of images to convert the color intensity of test line to green mode was performed using the free-access Inkscape 1.3.1 vector graphics editor (https://inkscape.org/, accessed on 16 December 2023).

### 3.3. Preparation of the AuNPs and the AuNP-Aptamer Conjugate

AuNPs were prepared following two different modified procedures based on the Frens method [27] and the Turkevich method [35] (for details see Supplementary Material). AuNPs were characterized by using UV/vis spectrophotometry and their average size was estimated based on the absorption maximum λ_max_ = 523 nm [36]. The AuNPs prepared using the modified Frens method had an average diameter of 22 nm, while those prepared with the modified Turkevich method had an average diameter of 14 nm (Figure S1, Supplementary Information).

The formation of aptamer-AuNP conjugates depends on the reaction between AuNPs and the sulfhydryl group of the thiol-modified aptamer to form Au-S bonds. The AuNP-aptamer conjugate was prepared based on a modified published procedure [27]. More specifically, 2 mL of AuNP solution was adjusted to pH 8.5 with 0.2 mol L^−1^ K_2_CO_3_ and centrifuged at 10,000 rpm for 30 min, the supernatant was discarded and the residue was re-suspended to 400 μL with molecular-biology-grade water. Afterwards, 10 μmol L^−1^ of the thiol-modified aptamer was mixed with AuNP solution at a volume ratio of 1:2 and left at room temperature for 8 h. Then, the mixture was salt-aged by adding 2 mol L^−1^ NaCl solution until a final concentration of 50 mmol L^−1^ was achieved and left for another 12 h. Finally, the mixture was centrifuged at 10,000 rpm for 30 min and the supernatant was discarded to remove unbound thiol-modified aptamer and AuNPs. The precipitate was redispersed in 10 mmol L^−1^ phosphate buffer (PBS) solution (pH 7.4) and stored at 4 °C.

### 3.4. Preparation of Streptavidin–Biotin–DNA Probe Conjugates for Test and Control Lines

The streptavidin–biotin DNA probe conjugates were formed by exploiting streptavidin–biotin binding, as reported previously [ 28]. The solution added to the test line was prepared as follows: streptavidin was dissolved into 10 mmol L^−1^ PBS (pH 7.4) solution to a final concentration of 1 mg mL^−1^. An amount of 35 μL of this solution was added to 35 μL of the 10 μmol L^−1^ probe 1 solution and incubated for 2 h at 4 °C, and then 30 μL of 10 mmol L^−1^ PBS (pH 7.4) was added into the mixture. Similarly, for the solution added to the control line, 35 μL of 1 mg mL^−1^ streptavidin solution and 35 μL of 20 μmol L^−1^ probe 2 were mixed and incubated for 2 h at 4 °C. The mixture was then added to 30 μL of 10 mmol L^−1^ PBS (pH 7.4). The streptavidin–biotin DNA probe conjugates were stored at 4 °C until their immobilization on the NC membrane.

### 3.5. Preparation of PVA Barrier Solution

An aqueous 1% *w*/*w* PVA solution was prepared by dissolving 0.1 g of PVA in 10 mL deionized water. Τhe solution was placed on a heating plate and stirred at 80 °C for 2 h to obtain a homogeneous solution. Finally, the solution was stored at 4 °C.

### 3.6. Preparation of the Lateral Flow Strip Components

The test line (T) and control line (C) of the aptasensor were drawn on the NC membrane by using an injection needle to dispense 2 μL solution of streptavidin–biotin–probe 1 and streptavidin–biotin–probe 2, respectively. The PVA barrier (B) was loaded onto the NC membrane upstream of the test line by dispensing 1.5 μL of 1% *w*/*w* PVA solution via an injection needle. The membranes were dried at room temperature for 2 h and stored at 4 °C until use.

The sample pad was soaked with 10 mmol L^−1^ PBS solution (pH 7.4) containing 1% *w*/w BSA and dried at 37 °C. The conjugate pad was soaked in 10 mmol L^−1^ PBS solution (pH 7.4) containing 1% *w*/*w* BSA, 0.25% *v*/*v* Tween 20 and 2% *w*/*w* sucrose and dried at 65 °C; then, 15 μL of the synthesized AuNP-aptamer conjugate solution was loaded on the conjugate pad and dried at 37 °C before use.

The consumables for each strip cost <0.10 in terms of reagents/materials.

### 3.7. Sample Preparation for OTA Detection

Beer, wine samples (white, rose and red), apple juice and milk with fat contents of 3.2% *w*/*v* and 0% *w*/*v* (zero-fat) were purchased from a local supermarket. Beer samples were degassed in an ultrasonic bath for 10 min and diluted 4 times with running buffer, prior to analysis. As for wine samples, the pH was adjusted from 3.3 to 7.0 using 2 mol L^−1^ NaOH. Then, white wine samples were diluted 10 times with running buffer, while rose and red wine samples were diluted 5 times with running buffer before analysis. pH adjustment to 7.0 was also carried out for apple juice samples using 2 mol L^−1^ NaOH and the samples were filtered through 0.20 µm filters and diluted with running buffer in a volume ratio 1:4. Finally, milk samples with 3.2% *w*/*v* fat content were centrifuged at 9500 rpm for 15 min, the upper fat layer was discarded and the lower milk layer was diluted with running buffer in a volume ratio of 1:20. Zero-fat milk samples were diluted 20 times with running buffer before analysis.

### 3.8. Detection of OTA

The detection of OTA was carried out by applying 50 μL of the appropriate OTA standard solution or sample to the sample pad. The solution was left to move across the strip for 10 min and then the strip was washed with 50 µL of running buffer, to remove any non-specifically adsorbed conjugates and other unretained sample components at the test and control lines. Readings (whether visual or colorimetric) were taken after 20 mins. The lateral flow strips were scanned with an HP Deskjet 2720 scanner (Hewlett Packard, Palo Alto, CA, USA) at high resolution (1200 dpi). Scanned images were processed by applying fluorescence and brilliance filters and the intensity of the color in the test line for each OTA concentration was converted to green mode (RGB mode) and measured using Inkscape. For calibration purposes, color intensities were graphically plotted against OTA concentrations. Τhe visual detection limit of the assay is defined as the minimum OTA concentration that can be visually differentiated from the blank solution. The quantitative LOD was determined from the calibration plot as the OTA concentration that produced a signal equal to −3 × SD (where SD is the standard deviation of the blank signal).

## 4. Conclusions

A fast, sensitive and low-cost colorimetric aptamer-based lateral flow assay was developed for OTA detection. The aptasensor achieves a visual LOD of 0.05 ng mL^−1^ and an instrumental LOD of 0.02 ng mL^−1^. The assay time is 20 min while other mycotoxins do not interfere. Τhe cost of each strip is calculated to be < 0.10 in terms of materials. The aptasensor was applied to the determination of OTA in beer, wine, apple juice and milk samples with recoveries in the range from 91 to 114% and relative standard deviations <14%. These biosensors are fit for purpose for the detection of OTA and compete favorably with existing aptasensing strips in terms of analytical features, simplicity, cost and speed.

## Figures and Tables

**Table 1:** Results of OTA determination in spiked samples obtained using the aptasensor developed in this work.

Sample	Amount Added (ng/mL)	Amount Determined (ng mL^−1^)	% Recovery	% RSD (*n* = 3)
Beer	0.1	0.104 ± 0.012	104	11.7
	1	0.95 ± 0.11	95	11.1
	10.0	10.50 ± 0.36	105	3.4
White wine	0.1	0.096 ± 0.011	96	11.8
	1	1.09 ± 0.04	109	3.7
	10.0	10.93 ± 0.85	109	7.8
Rose wine	0.1	0.104 ± 0.004	104	3.8
	1	0.937 ± 0.035	94	3.7
	10.0	9.8 ± 1.1	98	11.3
Red wine	0.1	0.090 ± 0.003	90	3.3
	1	0.977 ± 0.075	98	7.7
	10.0	9.07 ± 0.35	91	3.9
Apple Juice	0.1	0.114 ± 0.005	114	4.4
	1	1.12 ± 0.05	112	4.0
	10.0	9.45 ± 0.21	95	2.2
Milk (3.2% *w*/*v*)	0.1	0.094 ± 0.007	94	7.0
	1	0.95 ± 0.1	95	10.6
	10.0	9.07 ± 0.35	91	3.9
Milk (0% *w*/*v*)	0.1	0.105 ± 0.014	105	13.3
	1	1.13 ± 0.04	113	3.6
	10.0	9.69 ± 0.32	97	3.3

**Table 2:** Comparison of the assay proposed in this study with other reported methods for OTA detection using aptamer-based LFAs.

Detection	Labels	Visual LOD (ng/mL)	Quantitative LOD (ng/mL)	Linear Range	Total Assay Time (min)	Use of Activated Aptamer	Complex Preparation of Biorecognition Conjugate	Samples	Reference
Colorimetry	AuNPs	10^−4^	35 × 10^−6^	10^−4^–10^2^ ng/mL	200	Yes	Yes	Corn, rice, coffee bean	[26]
Colorimetry	AuNPs	1	0.18	0–2.5 ng/mL	<10	No	No	Red Wine	[27]
Visual	AuNPs	1	-	-	15	Yes	No	*Astragalus membranaceus*	[28]
Colorimetry	AuNPs	0.4	0.04	0–0.4 ng/mL	15	No	No	Red Wine	[29]
Colorimetry	AuNPs/ AgNPs	0.25	1.6	0.02–25.4 ng/mL	60	No	Yes	-	[30]
Fluorescence	Cy5	-	0.4	1–1000 ng/mL	20	No	No	Corn	[31]
Fluorescence	QDs	5	1.9	0–10 ng/mL	<15	No	No	Red Wine	[32]
Fluorescence	UCNPs	-	3	0.01–50 μg/mL	30	No	Yes	Real water	[33]
Fluorescence	UCNPs	-	1.86	5-100 ng/mL	15	Yes	Yes	Wheat, Beer	[34]
Colorimetry	AuNPs	0.05	0.02	0.05–25 ng/mL	20	No	No	Beer, Wine, Apple juice, Milk	This work

AuNPs: gold nanoparticles; AgNPs: silver nanoparticles; UCNPs: upconversion nanoparticles; QDs: quantum dots; Cy5: cyanine-5.

## Data Availability

Data provided in the Supplementary Materials.

## Supplementary Materials

The following supporting information can be downloaded at https://www.mdpi.com/article/10.3390/molecules28248135/s1, Preparation of the AuNPs; Figure S1: Absorption spectra of AuNPs with size of (a) 22 nm prepared using the modified Frens method and (b) 14 nm prepared using the modified Turkevich method; Figure S2. Selection of (a) the NC membrane, (b) the glass fiber conjugate pad and (c) the sample pad; Figure S3. Effect of (a) the type of buffer, (b) the concentration of PEG, (c) the Ficoll 400 concentration and (d) the Ficoll 70 concentration in the running buffer; Figure S4. Effect of the (a) the concentration of sucrose, (b) the concentration of BSA, (c) the type of surfactant, (d) the concentration of Tween in the running buffer and (e) the concentration of PVP to form the flow barrier.

## Abbreviations

OTA	ochratoxin A
AuNPs	gold nanoparticles
NC	nitrocellulose
IARC	International Agency for Research on Cancer
MLs	maximum limits
TLC	thin-layer chromatography
HPLC	high-performance liquid chromatography
GC	gas chromatography
SELEX	Systematic Evolution of Ligands by EXponential enrichment
AFB1	aflatoxin B1
FB1	fumonisin B1
DON	deoxynivalenol
PEG	polyethylene glycol
PVP	polyvinylpyrrolidone
PVA	poly vinyl alcohol
BSA	bovine serum albumin
RSD	relative standard deviation
LOD	limit of detection
QDs	quantum dots
AgNPs	silver nanoparticles
