# Supplementary material for: An Aptamer-Based Lateral Flow Biosensor for Low-Cost, Rapid and Instrument-Free Detection of Ochratoxin A in Food Samples
Source: Molecules. 2023 Dec 17;28(24):8135. doi: 10.3390/molecules28248135 (PMC10746035; doi:10.3390/molecules28248135)
Supplement: Supplementary file 1 [file molecules-28-08135-s001.zip › molecules-2782029-supplementary.pdf]

Supplementary Information

Development of an aptamer-based lateral flow biosensor for instrument-free rapid detection of ochratoxin A in food samples

Electra Mermiga[1], Varvara Pagkali[1], Christos Kokkinos[1], Anastasios Economou[1]

[1] Department of Chemistry, National and Kapodistrian University of Athens, 157 71 Athens, Greece

Preparation of the AuNPs

For the preparation of AuNPs based on the modified Frens method, all glassware was thoroughly cleaned with aqua regia solution (HCl/HNO_3, volume ratio 3:1) and was left to dry before use. Afterwards, 100 mL of 0.01% w/w $HAuCl_4$ solution was brought to boil and 1.8 mL of 1% w/w trisodium citrate solution was added rapidly under stirring. Then, the solution was boiled for 5 min and the color of the solution changed from blue to wine-red, indicating the formation of AuNPs. The solution was left to cool at room temperature and then, stored at 4°C in the dark.

According to the slightly modified Turkevich method, all glassware was firstly cleaned with aqua regia solution and was left to dry. Afterwards, 140 mL of 0.01% w/w $HAuCl_4$ solution was boiled vigorously under stirring, subsequently 14 mL of 38.8 mmol L^{-1} trisodium citrate solution was added and the mixture was allowed to boil until its color changed from pale yellow to the characteristic red color of AuNPs. After the formation of AuNPs, boiling of the solution was continued for another 10 minutes and then, the solution was left to cool at room temperature for 15 minutes. Finally, the solution was filtered through a 0.20 μm filter and stored at 4°C in the dark.

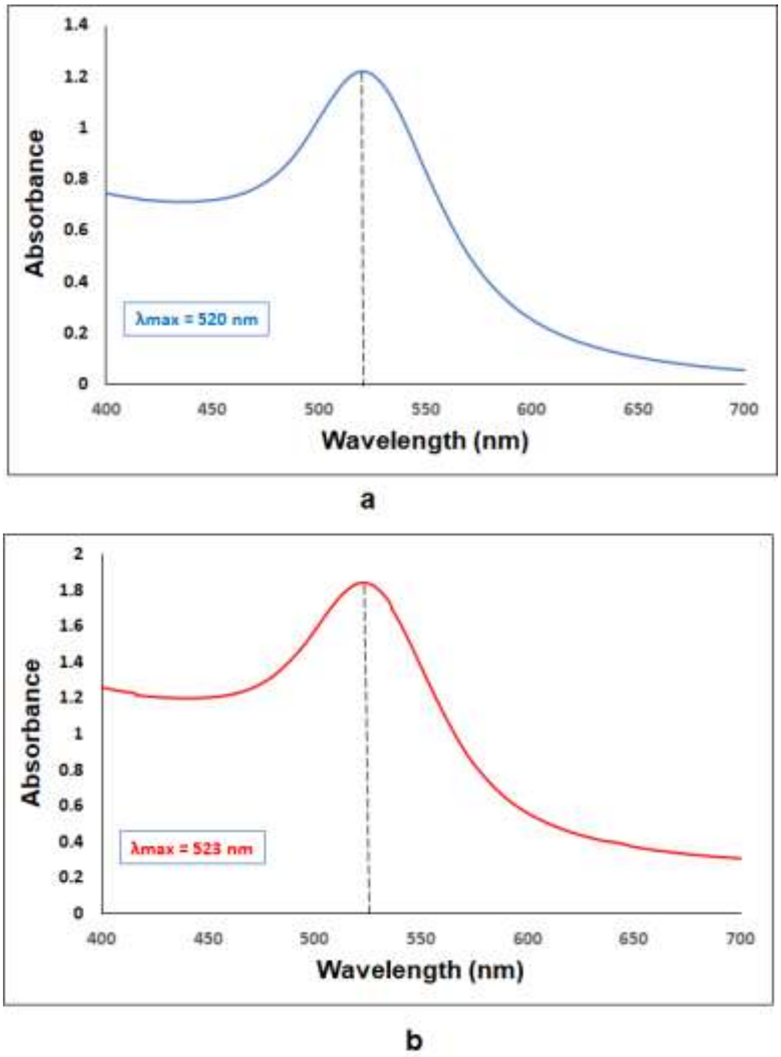

Figure S1. Absorption spectra of AuNPs with size of (a) 22 nm prepared using the modified Frens method; and, (b) 14 nm prepared using the modified Turkevich method.

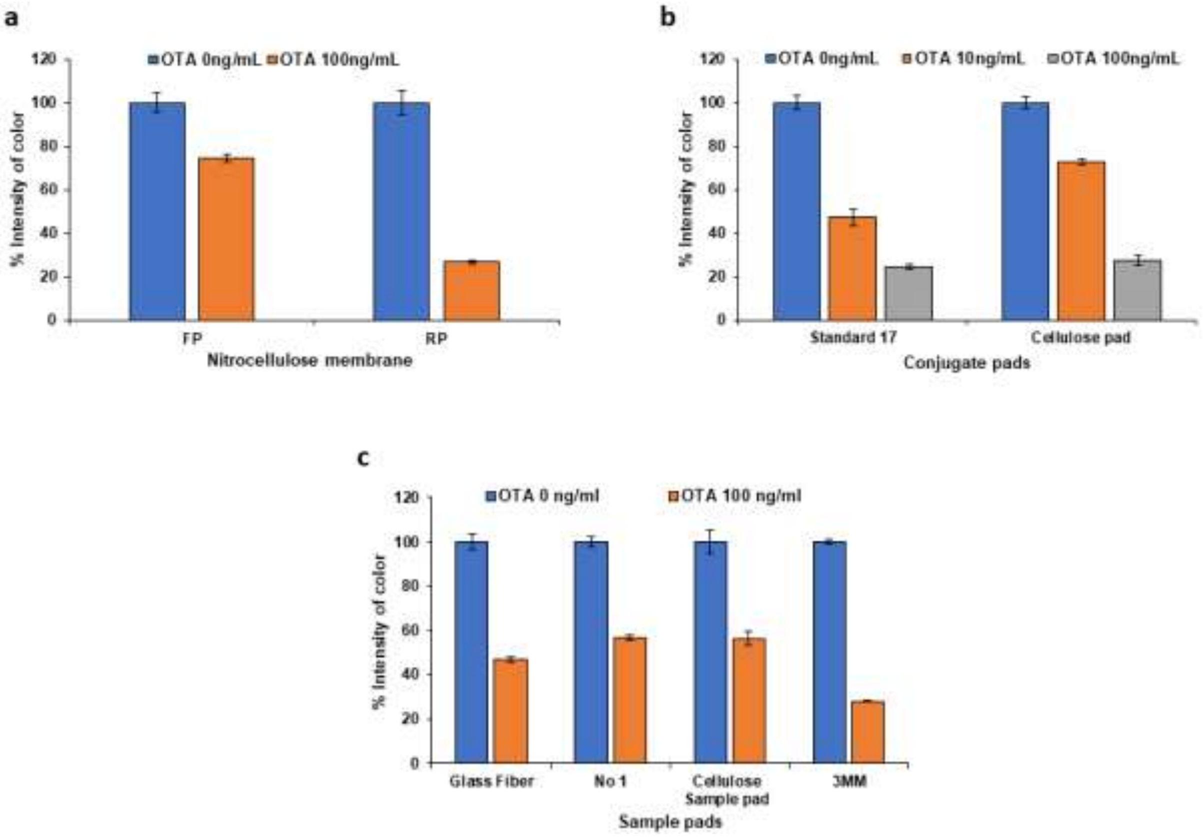

Figure S2. Selection of (a) the NC membrane, (b) the glass fiber conjugate pad and (c) the sample pad.

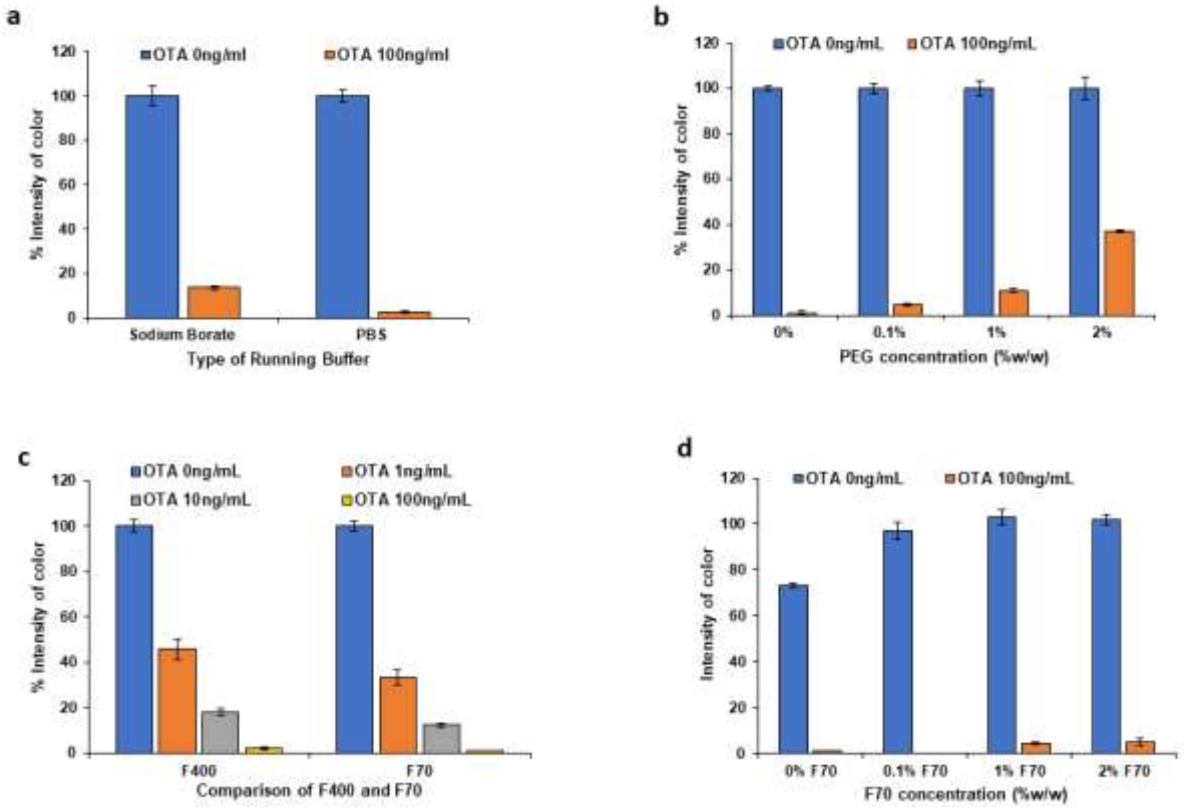

Figure S3. Effect of (a) the type of buffer, (b) the concentration of PEG, (c) the Ficoll 400 concentration and (d) the Ficoll 70 concentration in the running buffer.

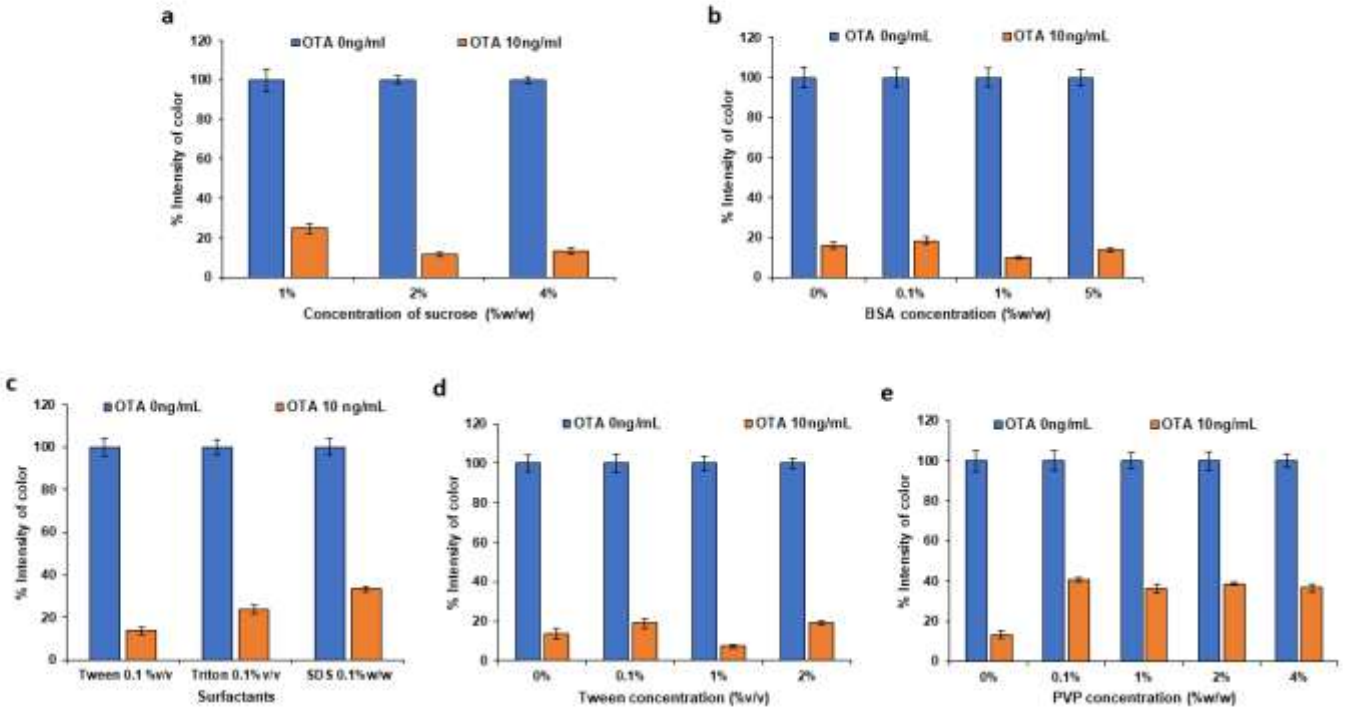

Figure S4. Effect of the (a) the concentration of sucrose, (b) the concentration of BSA, (c) the type surfactant (d) the concentration of Tween in the running buffer and (e) the concentration of PVP to form the flow barrier.
